# Testing undergraduate medical students’ ability to correctly identify skin conditions in skin of color—A pre-post-study at a medical school in Germany

**DOI:** 10.1371/journal.pone.0342922

**Published:** 2026-02-18

**Authors:** Finn Abeck, Ines Heinen, Isabel Heidrich, Julian Kött, Rachel Sommer, Christine Blome, Martin Härter, Matthias Augustin, Stefan W. Schneider, Inga Hansen-Abeck, Nina Booken

**Affiliations:** 1 Department of Dermatology and Venereology, University Medical Center Hamburg-Eppendorf, Hamburg, Germany; 2 Department of Medical Psychology, University Medical Center Hamburg-Eppendorf, Hamburg, Germany; 3 Institute for Health Services Research in Dermatology and Nursing (IVDP), University Medical Center Hamburg-Eppendorf, Hamburg, Germany; Independent Medical Researcher and Writer, UNITED KINGDOM OF GREAT BRITAIN AND NORTHERN IRELAND

## Abstract

**Background and objectives:**

Limited representation of diverse skin types in dermatology training hinders physicians’ ability to diagnose and treat patients with skin of color (SoC), thus contributing to health disparities. This study evaluated the effectiveness of a mandatory seminar in improving medical students’ ability to correctly identify skin conditions in SoC, as measured by objective tests.

**Methods:**

The pre-post design study was conducted among fourth year medical students at the University of Hamburg (Germany) between October 2024 and February 2025. A multiple-choice test using clinical images of eight SoC skin conditions assessed visual diagnostic skills at the beginning and end of a seminar.

**Results:**

The analysis included surveys from a total of 142 students (57.7% female, mean age: 25.2 years). The lowest pre-seminar identification rates were observed for melasma (26.8%) and keloids (40.1%). The overall identification rate improved significantly from 54.8% at the beginning of the seminar to 92.5% at the end of the seminar. The greatest increases in the proportion of correct diagnoses were found for melasma (+ 65.5%), keloids (+ 51.4%), and tinea (+ 47.9%). Furthermore, self-assessed knowledge on skin type classification scales and anatomical and physiological differences between light skin and SoC increased at the end of the seminar.

**Conclusion:**

The seminar effectively improved students’ ability to correctly identify skin conditions in SoC. Integrating SoC-focused training into medical curricula can bridge knowledge lacunae and thus help reduce health disparities by equipping future physicians in providing equitable care.

## Introduction

Skin conditions in people with skin of color (SoC, defined as Fitzpatrick skin phototypes IV-VI, which refer to a wide range of darkly pigmented skin [1]) are underrepresented in dermatology training [2,3], despite the specialty’s reliance on visual clinical diagnosis [4]. Inadequate knowledge of how dermatological conditions manifest themselves in SoC can lead to delays in diagnosis and poor or even incorrect treatment, contributing to health inequities [3,5,6]. Studies of both students and dermatologists show that skin conditions in SoC are less likely to be correctly identified than those on lighter skin [7,8]. Some skin diseases occur differently in light skin and SoC, like Melasma [9] or Keloids which occur significantly more often in patients with SoC [10]. In contrast, melanoma is less common in patients with SoC; however, in patients with SoC the disease is often already at a more advanced stage at the time of diagnosis [11].

The lack of diverse representation of skin types in dermatology training undermines the ability of future doctors to diagnose and treat patients with SoC [12]. Students attribute their lack of confidence in diagnosing skin conditions in patients with SoC to inadequate curriculum coverage [13]. Further research is needed to determine the most effective strategies for improving education about skin type diversity in medical training and to explore how these methods can support the development of skills needed to provide culturally competent care [14]. In 2022, around 1.27 million people with an African migrant background lived in Germany, reflecting the country's highly diverse society [15]. As diverse societies are a global phenomenon and people with SoC need to be diagnosed and treated correctly everywhere, it is important that students and physicians worldwide learn how to diagnose skin diseases in patients with SoC.

To address this lacuna in dermatology education in Germany, we implemented a seminar on skin type diversity as part of the regular medical curriculum at the University Medical Center Hamburg-Eppendorf, Germany in 2023 [16]. Preliminary evaluations based on student self-assessments showed an increase in self-reported competence in managing skin diseases in SoC, resulting from the seminar. In addition, most students indicated that they would like to attend more SoC courses in the future [16]. However, whether the seminar effectively improves ability to correctly identify skin conditions in SoC has not been evaluated to date. Our initiative aligns with international efforts to enhance diversity in dermatology education. The American Academy of Dermatology (AAD), among others, has launched dedicated programs and educational resources aimed at increasing the visibility of SoC in educational material and clinical training [17].

This study aimed to assess the effectiveness of the SoC seminar at the University Medical Center Hamburg-Eppendorf in improving medical students’ ability to correctly identify skin conditions in people with SoC, as measured by objective tests.

## Materials and methods

### Teaching format

The mandatory seminar was conducted in the fourth year of the 6-year medical curriculum (group size of approximately 20 students each), with the participation of the Department of Dermatology, Department of Medical Psychology, and Institute for Health Services Research in Dermatology and Nursing. Students are required to attend at least 85% of mandatory courses in undergraduate studies. This indicates that an attendance rate of less than 100% is possible, despite the seminar being compulsory. The interactive 90-minute seminar included a 45-minute session on skin-type diversity. It combined short lectures, case-based learning, and group discussions, and was aligned with cultural competence frameworks aimed at enhancing students’ awareness, knowledge, and skills in providing culturally competent dermatologic care. The session introduced the Fitzpatrick skin type classification system [1] and addressed the anatomical and physiological differences between lighter and darker skin. It also highlighted diagnostic challenges specific to SoC. Standardized clinical images were used to illustrate common skin conditions in SoC. The seminar incorporated psychosocial considerations and encouraged reflection on health equity in dermatological care [16].

### Pedagogical principles

The pedagogical principles applied in this seminar were based on Knowles’ adult learning theory [18], with a particular focus on the following aspects: (1) *the need to know*, as the importance of future diagnostic competences in SoC for patient-centered care was emphasized; (2) *experience*, as students discussed possible diagnoses in SoC during the seminar, drawing on their experiences from clinical internship or bedside teaching; (3) *orientation to learning*, as real-world images of patients were presented alongside an illustrated case report of a misdiagnosed patient with SoC; and (4) *motivation to learn*, as we expected students to be eager to enhance their knowledge of skin conditions in SoC and to improve their diagnostic abilities, leading to more accurate responses in the second multiple-choice test [18].

### Curriculum integration

In addition to the seminar presented here, the dermatology department held 13 lectures (each 45 minutes), as well as another seminar with interactive teaching and case studies on infectious diseases (90 minutes) and two bedside teaching sessions (each 90 minutes) in this module of medical studies. At the time of the seminar, at least 75% of the dermatology lectures in the curriculum had already been conducted; therefore, a certain level of dermatological knowledge among the students could be presumed. However, this was the first course focusing on SoC dermatology.

### Study design and questionnaire

The pre-post-survey via paper questionnaire was conducted between October 2024 and February 2025. The questionnaire was given to the students at the beginning of the seminar and the students were asked to choose the correct answer to the skin conditions presented by one photo for each condition via PowerPoint presentation for 20 seconds. The students had to choose in multiple-choice questions with one correct answer and five distractors, given the following instruction: “Please mark your suspected diagnosis”. At the end of the seminar, different pictures of the same skin conditions were shown again and the students were asked to choose the correct answer for the skin conditions, too.

In addition to general student characteristics (age, gender, current semester), students were asked about their previous dermatological experience at the beginning of the seminar. Interest in dermatology was measured on a six-point Likert scale (1 ‘very low interest’ to 6 ‘very high interest’), which was transformed into a three-level measurement, with low interest based on students answering 1 or 2, medium interest for students answering 3 or 4 and high interest for students answering 5 or 6.

### Skin conditions

The questionnaire on visual diagnosis contained 16 multiple-choice questions with clinical images (Fitzpatrick skin phototypes IV-VI [1]) without further information about the depicted patient of eight different skin conditions: tinea, melasma, atopic dermatitis, varicella, keloids, vitiligo, psoriasis and acral lentiginous melanoma. The diseases were selected to cover a wide range of dermatological conditions, including inflammatory, infectious and autoimmune diseases, as well as tumors. Additionally, melasma and keloids were chosen because they affect patients with SoC more frequently. Each picture was presented individually. All clinical images were standardized prior to the study to ensure consistent image quality. All images were reviewed by two independent, board-certified dermatologists to confirm diagnostic accuracy, ensure an accurate representation of SoC and typical efflorescences, and ensure a comparable level of difficulty across cases. Within the seminar, the eight skin diseases were presented and diagnostic criteria were explained in detail, but different photos were used for the illustrations of the individual skin diseases. Therefore, different photos were used in the multiple-choice tests and in the seminar.

### Students’ self-assessment

The ability to make a dermatological diagnosis in patients with SoC, the students’ knowledge of skin type scales and the students’ knowledge of anatomical and physiological differences between light skin and SoC were assessed according to self-assessment at the beginning and end of the seminar. Ratings were made according to a Likert scale ranging from 1 to 6 (ability/knowledge ’very low’ to ’very high’). As this 6-point-Likert scale was based on regular teaching evaluations of the medical degree program, students were already familiar with it from previous evaluations [19].

### Ethical considerations and participants

The study design was reviewed and approved by the local Psychological Ethics Committee of the Center for Psychosocial Medicine (LPEK-0688). Prior to the survey, the students were provided with an information sheet regarding the study. Survey participation was voluntary and anonymous. The participants did not receive any compensation. Nonparticipation in the survey resulted in no negative consequences for students. Written informed consent was obtained from all participants.

### Statistical analysis

Data were analyzed using GraphPad Prism version 8 (GraphPad Software, San Diego, CA, USA) and IBM SPSS Statistics version 29. Differences in the number of correctly rated visual diagnoses between the pre- and post-seminar answers were analyzed using McNemar tests for paired, nominal data. The means of the students’ self-assessed skills and knowledge were compared using paired *t*-tests. Statistical correlation was measured using Pearson's correlation coefficient. Statistical tests were two-tailed, and the α-level of 5% was set at *p* < 0.0042 after Bonferroni adjustment for multiple testing. As the seminar was mandatory and the seminar’s content had to be the same for all student groups it was impossible to include a control group.

## Results

### General students’ characteristics

Out of a total of 172 students in the semester, 151 students attended the seminar (87.8%). The participation rate in the voluntary questionnaire survey was 94.0% (*n* = 142). Of the students, 57.7% were female (*n* = 82; representing the current gender distribution in medical studies at the University of Hamburg [20]) and the average age was 25.2 ± 2.6 years (range 21–35 years). 92.3% (*n* = 131) of the students were in their 7th semester. A high interest in dermatology was confirmed by 21.8% (*n* = 31) of the participants (Table 1).

**Table 1:** General characteristics of the 142 students.

	Students *n* = 142
**Age**	
Mean value (SD)	25.2 (2.6)
Range	21–35
**Sex, *n* (%)**	
Female	82 (57.7)
Male	60 (42.3)
**Semester, *n* (%)**	
Six	5 (3.5)
Seven	131 (92.3)
Eight	4 (2.8)
Nine	2 (1.4)
**Interest in Dermatology, *n* (%)**	
Low	35 (24.6)
Medium	76 (53.5)
High	31 (21.8)
**Previous dermatological experience, *n* (%)**	
Yes	14 (9.9)
No	128 (90.1)

SD: standard deviation.

### Students‘ self-assessment

**Fig 1:**
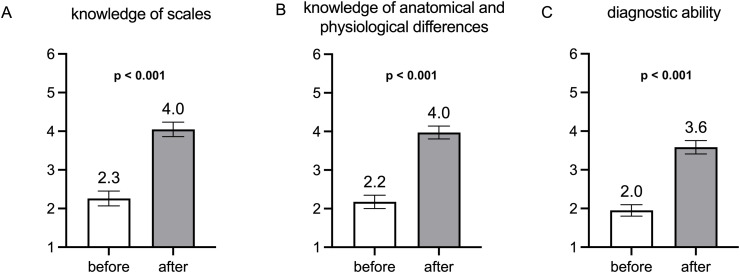
Means and 95% confidence interval of the students’ self-assessment (*n = 142*); survey before and after the seminar. **A)** Assessment of the knowledge of scales for classification of different skin types. **B)** Knowledge of the anatomical and physiological differences between light skin and SoC (**C)** Assessment of own ability to identify skin diseases in people with SoC.

At the beginning of the seminar, 23.2% (*n* = 33) of the students stated they were familiar with skin type classification scales. There was a significant increase in self-reported knowledge of skin type scales at the end of the seminar (2.3 ± 1**.**1 vs. 4.0 ± 1.1, *p* < 0.001; Likert scale from 1 ‘very low knowledge’ to 6 ‘very high knowledge’; Fig 1A). The students’ self-assessment of their knowledge of the anatomical and physiological differences between light skin and SoC was 2.2 ± 1.1 on average (scale from 1 ‘very little knowledge’ to 6 ‘very high knowledge’). After attending the seminar, students rated their knowledge significantly higher than before (4.0 ± 1.0; *p* < 0.001; Fig 1B).

### Identification of skin diseases in patients with SoC

**Fig 2:**
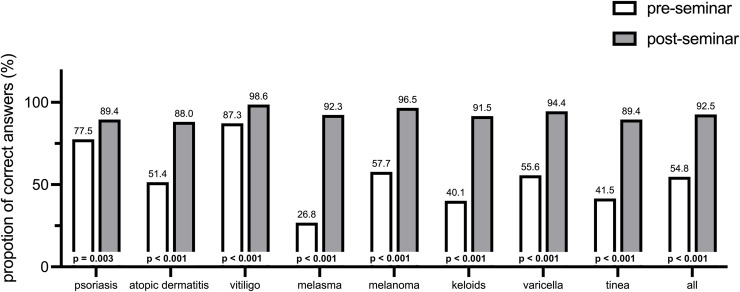
Percentage of students that accurately identified each skin condition in skin of color pre-seminar and post-seminar *(n = 142)* as well as the *p*-values of the individual McNemar-tests.

At the beginning of the seminar, the percentage of accurately identified visual diagnoses was 54.8% (M = 4.4 correct diagnoses). The diagnoses with the lowest percentage of correct answers at the beginning of the seminar were melasma (26.8%) and keloids (40.1%). Vitiligo was correctly diagnosed by 87.3% of the students. Students with a high interest in dermatology showed a higher number of correctly identified visual diagnoses at the beginning of the seminar than students with a medium to low interest in dermatology (68.1% vs. 51.0%, *p* < 0.001). At the end of the seminar, there was a significant increase in the number of correct diagnoses which rose to an average of 92.5% (M = 7.4 correct diagnoses, *t*-*t*est for paired samples: *t*(141)=−21.732, *p* < 0.001, Cohen’s d = 1.66). The grea*t*est increases in correct diagnoses were shown for melasma (+65.5% correct answers), keloids (+51.4%) and tinea (+47.9%; Fig 2).

Self-assessed ability to diagnose skin diseases in SoC also increased significantly from 2.0 ± 0.9 (Likert scale from 1 ‘very low ability’ to 6 ‘very high ability’) at the beginning of the seminar to 3.6 ± 1.1 at the end of the seminar (*p* < 0.001; Fig 1C). No significant correlation was found between overall performance on the multiple-choice test and self-assessed diagnostic ability at the beginning (r = 0.10, *p* = 0.221). At the end of the seminar, performance on the multiple-choice test and self-assessed diagnostic ability showed a very weak positive correlation (r = 0.20, *p* = 0.020).

## Discussion

Due to the lack of diverse representation of skin types in dermatology training, visual diagnosis of skin conditions in SoC is a challenge and a potential cause of misdiagnosis in clinical practice [7]. As the diversity of skin types in the patient population continues to increase, research into the effectiveness of newly developed modules/seminars is crucial to improving the medical curriculum. Our study demonstrated that the implementation of a specific SoC dermatology seminar increased the ability of medical students to correctly identify skin diseases in SoC on a both statistically and clinically significant level. For all eight dermatological conditions studied, there was an improvement in the students’ identification rates at the end of the seminar. Unlike our previous study [16], the improvement was not based on self-report alone but was objectified by a multiple-choice test. Our teaching methods included parts of perceptual learning. Perceptual learning is a change of perception as a result of experience. Interpreting visual findings is a core goal of dermatology education. Recognizing dermatologic conditions largely depends on implicit, non-analytical knowledge developed through exposure to images and patient cases [21,22].

The two diagnoses with the lowest proportion of correct answers in the pre-seminar test were melasma (26.8%) and keloids (40.1%), even though melasma is one of the most common hyperpigmentation skin disorders and occurs more commonly in SoC [9]. Keloids also occur significantly more often in patients with SoC [10]. Disparities in dermatologic health care disproportionately impact patients with SoC, leading to misdiagnoses and delayed treatment [23,24]. Among the various factors contributing to these disparities, one physician-dependent factor is the limited understanding of disease presentations in patients with SoC, which negatively affects the quality of care provided [25,26]. In our study, the number of correct diagnoses improved after the SoC seminar for both conditions, melasma (92.3%) and keloids (91.5%).

While melasma and keloids are bothersome but benign skin conditions, melanoma is a highly malignant tumor. While melanoma is less common in patients with SoC, those who are diagnosed often have more advanced disease [11]. This disparity is partly due to differences in the types of melanoma that predominantly affect patients with SoC, such as acral lentiginous melanoma. This type of melanoma is not associated with UV exposure and may be overlooked in clinical assessments that typically focus on UV-related risk factors [27]. In our study, the percentage of students who could correctly identify acral lentiginous melanoma at the end of the SoC seminar was 96.5%, whereas at the beginning of the seminar only 57.7% students rated this visual diagnosis correctly. To improve the ability of physicians to diagnose melanoma in SoC, dermatology education in medical schools needs to cover the differences in risk factors and a broader spectrum of clinical presentation of melanoma in different skin types [28].

Our findings demonstrate the great importance of incorporating SoC education into standard curricula to reduce health disparities. Furthermore, knowledge of skin type scales and of the anatomical and physiological differences between fair skin and SoC improved according to the students’ self-assessment. Although dermatology relies on visual diagnosis, a fundamental understanding of skin structure and function is crucial for managing skin diseases. For example, in patients with SoC, the risk of post-inflammatory hyperpigmentation is higher due to larger melanosomes and increased melanin content, while the likelihood of keloid formation is greater due to numerous, large, and multinucleated fibroblasts [29].

The skin diseases with the highest proportion of correct answers in the pre-seminar test were vitiligo (87.3%) and psoriasis (77.5%) – even before they were explained in detail during class. Vitiligo is a very characteristic skin condition and became known worldwide by the model Winnie Harlow and the singer Michael Jackson [30], which may explain the high number of correctly identified diagnosis in the pre-test. With a prevalence of around 2%, psoriasis is one of the most common chronic skin diseases in Germany, which may have contributed to the high identification rates [31]. Although both conditions were often correctly diagnosed in the pre-test, an improvement in the ability to correctly identify vitiligo and psoriasis was observed after the seminar.

Previous research has investigated the implementation of dermatology courses focusing on SoC for medical students, but with less participants than in our study and in voluntary classes. Arza et al. [13] conducted a voluntary virtual seminar for medical students with 70 attendees, focusing on skin health equity and education on skin diseases prevalent in SoC. After the seminar, most students reported lack of confidence about their ability to diagnose skin conditions in SoC and most rated institutional learning resources for skin diversity education as inadequate [13].

Shango et al. [32] offered a voluntary supplemental module with 77 attendees as part of a second-year dermatology curriculum, which comprised thirteen cases of common skin conditions in African American patients. After completing the module, the medical students reported a significant increase in confidence in diagnosing skin conditions in SoC, too [32].

Han et al. [8] developed a virtual lecture for medical students covering skin diseases of all skin types. The study of 43 participants showed an improvement of 30.1% in correct visual diagnosis among 9 skin conditions in patients with SoC. The most significant improvements were seen in psoriasis vulgaris (54.9%), rosacea (47.3%) and seborrheic keratosis (40.6%). In addition, the authors showed that on the pre-examination questionnaire, students scored lower for diagnosing skin diseases in patients with SoC than for patients with a lighter skin.

However, unlike the SoC seminar we developed, participation in the courses of Han et al. [8], Shango et al. [32] and Arza et al. [13] was voluntary and therefore not integrated into the regular curriculum. In our study cohort, only 21.8% of students reported a high interest in dermatology, whereas the participants in the three studies mentioned before were probably very interested in dermatology to attend a voluntary class. As our seminar was compulsory, even students with a moderate or low interest in the field attended the SoC seminar. Therefore, our results demonstrate that targeted seminars can improve medical student’s ability to correctly identify skin conditions in SoC regardless of students’ personal interest in the subject. Given that the treatment of dermatological conditions such as atopic dermatitis and skin cancer screening is not limited to dermatologists [33,34], a thorough understanding of skin diseases in different skin types is highly relevant for all future physicians. Comparable studies have shown the positive effects of SoC programs also for dermatology residents [35] and physician assistants [36].

A strength of our study is the robust assessment of diagnostic skills using a multiple-choice test at the beginning and the end of the seminar. As the seminar was integrated into the regular, mandatory curriculum, all students attended regardless of their interest in dermatology, resulting in a large study cohort. The study is limited by its quasi-experimental pre-post design without a control group, which does not allow causal conclusions to be drawn. Additionally, the results could have been influenced by potential confounding factors such as students’ prior dermatological experience, baseline knowledge and varying levels of interest in dermatology. The multiple-choice test was administered immediately after the seminar. This immediate post-seminar assessment provides valuable insight into short-term learning effects but does not allow conclusions about sustained knowledge retention or diagnostic performance over time. Future research should include follow-up assessments after several months to evaluate the durability of learning outcomes and the long-term impact of SoC education on students’ diagnostic ability. Multiple-choice questions listing diseases do not accurately reflect real-life scenarios. To improve the validity of assessments, future studies should use standardized real-life cases. The study was conducted in just one single university hospital in Germany, which limits the generalizability of the findings to other geographical areas. The transferability of the results may be affected by cultural and institutional variables. Differences in medical education curricula and in the representation of diverse populations across countries could influence how similar teaching formats are perceived and how effective they are in improving students’ ability to correctly identify skin diseases. Further research in diverse educational and cultural settings is needed to examine the broader applicability of such interventions.

## Conclusion

This study highlights the importance of incorporating SoC training into medical curricula to reduce health disparities by equipping future physicians in providing equitable care. We strongly recommend further research to determine best practices for the sustainable integration of SoC dermatology within medical school education, including studies with long-term follow up.

## Supporting information

S1 File Data set. (XLSX)
